# Development of a Clinicopathological Prognostic Model and Risk Classification to Predict Disease-Free Survival in Patients with Gastric Adenocarcinoma Following Neoadjuvant Chemotherapy and Curative Gastrectomy

**DOI:** 10.3390/curroncol33090500

**Published:** 2026-08-24

**Authors:** Erdoğan Şeyran, Emre Hafızoğlu

**Affiliations:** 1 Van Training and Research Hospital, University of Health Sciences, Van 65300, Türkiye; 2 Department of Medical Oncology, Bursa City Hospital, Bursa 16110, Türkiye; emre.hafizoglu@saglik.gov.tr

**Keywords:** Becker tumor regression grade, disease-free survival, gastric adenocarcinoma, neoadjuvant chemotherapy, prognostic model, risk classification

## Abstract

Background: Prognostic assessment after neoadjuvant chemotherapy and curative gastrectomy remains challenging in patients with gastric adenocarcinoma because postoperative outcomes are influenced by both pretreatment disease burden and pathological response. We aimed to develop and internally validate a clinicopathological prognostic model and a simple postoperative risk classification for predicting disease-free survival (DFS). Methods: This single-center retrospective cohort study included patients with gastric adenocarcinoma who underwent neoadjuvant chemotherapy followed by curative gastrectomy. Pretreatment clinicopathological variables, Becker tumor regression grade (TRG), and serum tumor markers were evaluated. Logistic regression was used to identify predictors of favorable pathological response, whereas Cox proportional hazards regression was performed to identify independent prognostic factors for disease-free survival (DFS). Sequential prognostic models were developed and internally validated using 1000 bootstrap resamples. A simplified postoperative clinicopathological risk classification based on pretreatment clinical N stage and Becker tumor regression grade was additionally developed to facilitate clinical interpretation and postoperative risk stratification. Results: A total of 109 patients were included. Favorable pathological response (Becker TRG1–2) was achieved in 68 patients (62.4%), whereas 41 patients (37.6%) had minimal or no pathological response (TRG3). In multivariable logistic regression analysis, pretreatment clinical T stage (cT4 vs. cT1–3) and clinical N stage (cN2–3 vs. cN0–1) were independently associated with a lower likelihood of achieving a favorable pathological response. For disease-free survival, pretreatment clinical N stage, Becker tumor regression grade, and log10-transformed CA19-9 remained independent prognostic factors in the multivariable Cox model. Sequential model development demonstrated progressive improvement in model discrimination, with the optimism-corrected Harrell’s C-index increasing from 0.697 for the clinical N stage model to 0.770 for the final model incorporating clinical N stage, Becker tumor regression grade, and CA19-9. Bootstrap internal validation demonstrated minimal optimism, and calibration analysis showed good agreement between predicted and observed disease-free survival. A simple postoperative clinicopathological risk classification successfully stratified patients into distinct prognostic groups. Conclusions: A clinicopathological prognostic model integrating pretreatment clinical N stage, Becker tumor regression grade, and serum CA19-9 demonstrated improved prognostic discrimination for disease-free survival compared with clinical N stage alone. The derived postoperative risk classification may provide a simple framework for postoperative risk stratification and could assist in individualizing postoperative surveillance. External validation is warranted before routine clinical implementation.

## Simple Summary

Patients with locally advanced gastric cancer are commonly treated with chemotherapy before surgery, but the risk of cancer recurrence after surgery varies considerably among patients. Identifying patients at higher risk of recurrence may help improve postoperative follow-up and guide future treatment strategies. In this study, we evaluated clinical, pathological, and blood-based factors that are routinely available in patients who received chemotherapy followed by curative gastrectomy. We found that the extent of lymph node involvement before treatment, the degree of tumor regression after chemotherapy, and the serum tumor marker carbohydrate antigen 19-9 provided complementary prognostic information for disease-free survival. Combining these factors improved the ability to distinguish between patients with different outcomes. We also developed a simple risk classification based on pretreatment lymph node status and pathological tumor regression. These findings may support more individualized postoperative risk assessment and surveillance, although validation in independent patient populations is required before routine clinical use.

## 1. Introduction

Gastric adenocarcinoma remains a major global health challenge and continues to be a leading cause of cancer-related morbidity and mortality despite substantial advances in multimodal treatment. For patients with resectable locally advanced disease, neoadjuvant chemotherapy followed by curative gastrectomy has become the standard treatment strategy according to current international guidelines [1,2]. Accurate pretreatment diagnosis and staging are fundamental to treatment planning, with contrast-enhanced computed tomography and histopathological examination of endoscopic biopsy specimens forming key components of the diagnostic work-up. Taxane-containing perioperative or neoadjuvant chemotherapy represents an important component of multimodal treatment for locally advanced gastric cancer. In addition to the widely adopted FLOT regimen, docetaxel-, oxaliplatin-, and capecitabine-based chemotherapy has demonstrated feasibility and pathological activity in patients with clinical T3–T4 non-metastatic gastric cancer [3]. More recently, the phase III MATTERHORN trial demonstrated that the addition of durvalumab to perioperative FLOT significantly improved event-free survival and increased the pathological complete response rate compared with FLOT alone in patients with resectable gastric or gastroesophageal junction adenocarcinoma [4]. Nevertheless, recurrence remains frequent after curative-intent treatment, and postoperative outcomes vary considerably even among patients with similar clinicopathological characteristics, underscoring the need for more precise postoperative risk stratification [2,5].

Pathological tumor regression after neoadjuvant chemotherapy is widely recognized as an indicator of treatment response and tumor biology in gastric adenocarcinoma. Among the available regression systems, the Becker tumor regression grade (TRG) is routinely used to quantify residual viable tumor after preoperative therapy [6]. Previous studies have consistently shown that favorable tumor regression is associated with improved disease-free and overall survival [7,8]. Nevertheless, patients within the same TRG category may experience markedly different postoperative outcomes, indicating that pathological response alone cannot fully explain recurrence risk. Integrating TRG with complementary clinicopathological factors may therefore improve postoperative prognostic assessment.

Recurrence risk after neoadjuvant chemotherapy and curative gastrectomy varies substantially among patients and is unlikely to be captured by a single clinicopathological factor. Pretreatment clinical nodal (cN) stage provides information on the initial extent of disease and has been associated with recurrence and survival following multimodal treatment [9,10]. Serum carbohydrate antigen 19-9 (CA19-9) may add further prognostic information, as higher levels have been linked to unfavorable outcomes [11]. In contrast, pathological tumor regression reflects the response of the primary tumor to neoadjuvant treatment. Despite representing different aspects of the disease course, these factors are often assessed separately, and evidence regarding their combined value for predicting disease-free survival remains limited.

Several prognostic models have been proposed for gastric cancer, but most were developed in patients undergoing upfront surgery or rely primarily on postoperative pathological staging [12]. More recent prognostic models have integrated clinicopathological characteristics, pathological response, lymph node status, and other disease-related variables to improve individualized survival and recurrence risk estimation [12,13,14]. However, practical models integrating pretreatment clinical nodal stage, Becker tumor regression grade, and routinely available serum biomarkers for individualized prediction of disease-free survival after neoadjuvant chemotherapy remain scarce.

Therefore, this study aimed to develop and internally validate a clinicopathological prognostic model for predicting disease-free survival in patients with locally advanced gastric adenocarcinoma treated with neoadjuvant chemotherapy followed by curative gastrectomy. In addition, we developed a simplified postoperative risk classification based on pretreatment clinical nodal stage and Becker tumor regression grade to facilitate individualized postoperative risk stratification.

## 2. Materials and Methods

### 2.1. Study Design and Patient Population

This retrospective single-center cohort study included consecutive patients with locally advanced gastric adenocarcinoma who underwent neoadjuvant chemotherapy followed by curative-intent gastrectomy at the Department of Medical Oncology, Van Training and Research Hospital, University of Health Sciences, Van, Türkiye, between January 2015 and January 2025. Eligible patients were identified through institutional electronic medical records, pathology reports, surgical records, and the institutional oncology database.

**Figure 1:**
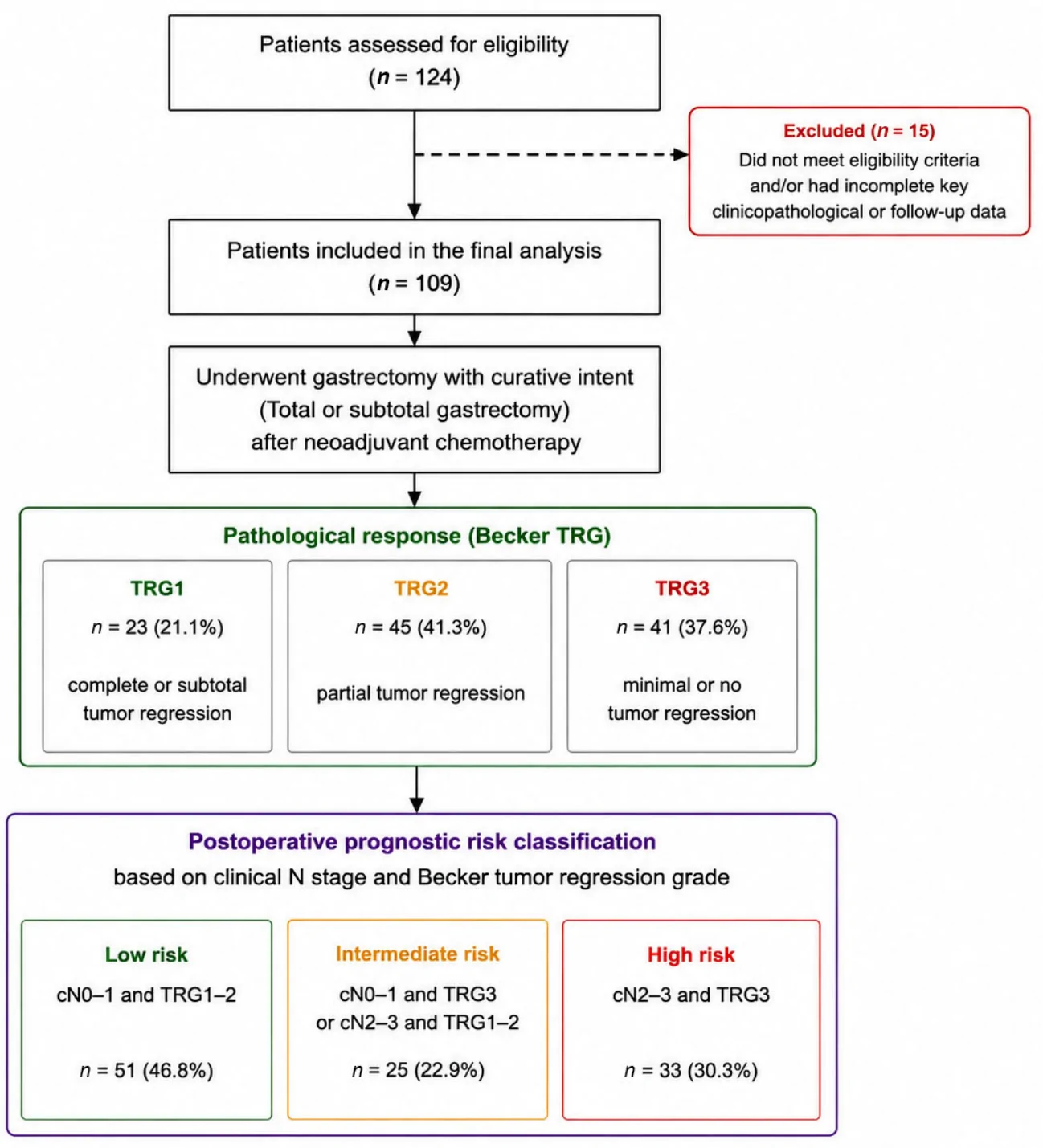
Study flow diagram. TRG, Becker tumor regression grade; cN, clinical nodal stage.

Patients were eligible if they had histologically confirmed gastric adenocarcinoma, received neoadjuvant chemotherapy followed by curative-intent gastrectomy, and had complete data for the key variables required for the present analyses, together with adequate follow-up information. Patients with metastatic disease at diagnosis, non-adenocarcinoma histology, previous or synchronous malignancies that could influence survival assessment, palliative surgery, incomplete pathological evaluation, or missing key clinical data required for prognostic model development were excluded. The patient selection process is summarized in Figure 1.

### 2.2. Data Collection

Baseline demographic, clinical, laboratory, treatment, and pathological data were retrospectively collected from the institutional electronic medical records and hospital information system. Recorded variables included age, sex, Eastern Cooperative Oncology Group performance status, tumor location, pretreatment clinical T and N stage, neoadjuvant chemotherapy regimen, pretreatment serum CA19-9, type of gastrectomy, tumor differentiation, lymphovascular invasion, perineural invasion, resection margin status, lymph node yield, and Becker tumor regression grade. Pathological T and N categories were additionally recorded when clearly documented; however, standardized ypTNM staging was not consistently available for all patients throughout the study period. Before initiation of neoadjuvant treatment, clinical staging was based on contrast-enhanced computed tomography, upper gastrointestinal endoscopy, and histopathological examination of endoscopic biopsy specimens. Clinical T and N stages were assigned according to the 8th edition of the American Joint Committee on Cancer (AJCC) TNM staging system. Helicobacter pylori or other microbiological testing was not systematically available in the retrospective records and was therefore not included in the present analysis. Variables used in the survival analyses were obtained from the medical records before the statistical analyses were performed.

### 2.3. Neoadjuvant Treatment and Surgical Procedures

Neoadjuvant chemotherapy was administered according to contemporary institutional treatment protocols and multidisciplinary tumor board recommendations. Treatment regimens were selected at the discretion of the treating physician based on patient performance status, comorbidities, and prevailing clinical practice. Following completion of neoadjuvant chemotherapy, patients were re-evaluated by clinical examination and radiological imaging to determine eligibility for curative-intent surgery. Eligible patients underwent subtotal or total gastrectomy with regional lymphadenectomy according to tumor location and disease extent. All surgical procedures were performed by experienced gastrointestinal surgeons. Gastrectomy was performed using either an open or laparoscopic approach according to tumor characteristics, surgical assessment, and institutional practice; no robotic procedures were performed during the study period. Standard D2 lymphadenectomy represented the predominant lymphadenectomy approach. Detailed information on individual lymph node yield and distinctions between D2-plus and D3 lymphadenectomy was not consistently available in the retrospective records and therefore could not be systematically reported. Resected specimens were evaluated by specialized gastrointestinal pathologists using standardized institutional protocols, and pathological findings were documented in the final pathology reports.

### 2.4. Histopathological Evaluation

Surgical specimens were reviewed by gastrointestinal pathologists as part of routine pathological evaluation. Recorded pathological features comprised histological type and differentiation, pT and pN categories, lymphovascular and perineural invasion, resection margin status, and the numbers of examined and metastatic lymph nodes. Pathological staging was based on the 8th edition of the AJCC TNM classification in cases with adequate information for stage assignment.

Tumor response to neoadjuvant chemotherapy was assessed using the Becker tumor regression grading (TRG) system. Tumor regression was classified as TRG1a (complete regression, no residual tumor cells), TRG1b (subtotal regression, <10% residual tumor), TRG2 (partial regression, 10–50% residual tumor), and TRG3 (minimal or no regression, >50% residual tumor). For statistical analyses, TRG1a, TRG1b, and TRG2 were grouped as a favorable pathological response, whereas TRG3 was classified as an unfavorable pathological response. For survival analyses, TRG1a and TRG1b were combined and analyzed as TRG1, whereas TRG2 and TRG3 were retained as separate categories.

### 2.5. Follow-Up and Study Endpoints

Patients were followed from the date of curative gastrectomy until disease recurrence, death, or the last available follow-up. Postoperative surveillance was performed according to institutional practice and current clinical guidelines, including regular clinical assessments, laboratory investigations, and contrast-enhanced computed tomography at predefined intervals. Disease recurrence was confirmed by radiological, endoscopic, or histopathological findings when available. Disease-free survival (DFS) was defined as the interval between curative gastrectomy and the first documented recurrence or death from any cause. Patients without an event were censored at the date of the last follow-up.

### 2.6. Development of the Prognostic Model and Risk Classification

A clinicopathological prognostic model for disease-free survival (DFS) was developed using the independent prognostic factors identified in the final multivariable Cox proportional hazards model. Sequential model development was performed to evaluate the incremental prognostic value of individual variables. Model 1 included pretreatment clinical N (cN) stage alone, Model 2 incorporated cN stage and Becker tumor regression grade (TRG), and Model 3 additionally included log10-transformed serum carbohydrate antigen 19-9 (CA19-9). Model performance was evaluated using Harrell’s concordance index (C-index), likelihood-ratio chi-square statistics, and bootstrap internal validation with 1000 resamples. Calibration of the final model was assessed using bootstrap-corrected calibration plots at 24 months after surgery. The 24-month time point was selected because it closely approximated the median disease-free survival and was supported by adequate follow-up within the study cohort. Calibration at additional time points was not formally evaluated because 24 months was selected as the primary clinically relevant landmark for this analysis, while later time points were associated with progressively fewer patients remaining at risk and potentially less stable estimates. Observed 24-month disease-free survival probabilities were estimated using Kaplan–Meier jackknife pseudo-values and compared with model-predicted probabilities across quintiles of predicted risk. Optimism correction and 95% confidence intervals for the calibration curve were obtained using 1000 bootstrap resamples.

To facilitate clinical interpretation, a simplified postoperative clinicopathological risk classification was subsequently developed using pretreatment clinical N stage and Becker tumor regression grade, which represented the two strongest clinicopathological predictors of disease-free survival. Patients were classified into three clinically defined risk groups: low risk (cN0–1 and TRG1–2), intermediate risk (cN0–1 and TRG3 or cN2–3 and TRG1–2), and high risk (cN2–3 and TRG3). The discriminatory ability of the proposed risk classification was evaluated using Kaplan–Meier survival analysis and the log-rank test. To enhance model transparency and reproducibility, all predictors included in the final prognostic model were defined using routinely available clinical, pathological, and laboratory variables, and their coding and transformations are explicitly reported. The simplified risk classification was derived from combinations of clinical N stage and Becker tumor regression grade and does not require specialized software for application. Consistent with current prediction-model reporting principles, the present model should be considered a development and internal-validation model; independent external validation is required before its use for individual clinical decision-making.

### 2.7. Statistical Analysis

Statistical analyses were conducted with IBM SPSS Statistics for Windows, version 25.0 (IBM Corp., Armonk, NY, USA). R software, version 4.6.0 (R Foundation for Statistical Computing, Vienna, Austria) was additionally used for model performance assessment and internal validation, including calculation of Harrell’s concordance index (C-index), bootstrap-based optimism correction, and calibration. Distributional characteristics of continuous variables were examined using the Shapiro–Wilk test. Continuous data are summarized as mean ± standard deviation (SD) or median (range), depending on their distribution, while categorical data are reported as numbers and percentages. For comparisons between groups, the independent-samples *t*-test or Mann–Whitney U test was applied to continuous variables, whereas categorical variables were compared using the chi-square test or Fisher’s exact test, as appropriate.

Factors associated with favorable pathological response were investigated using logistic regression in univariable and multivariable analyses. Disease-free survival (DFS) was analyzed using the Kaplan–Meier method, and differences between survival curves were evaluated using the log-rank test. Associations between candidate variables and DFS were first examined using univariable Cox proportional hazards regression. The proportional hazards assumption was evaluated graphically using log-minus-log survival plots, without evidence of major violations. Analyses were based on patients with available data for the variables required for the respective analyses and adequate follow-up information; missing values were not imputed. Variables with *p* < 0.10 in univariable analysis were considered for multivariable Cox regression together with prespecified clinically relevant covariates, namely histological grade, clinical T stage, clinical N stage, treatment regimen, Becker tumor regression grade, CEA, CA19-9, and albumin. The independent predictors retained after multivariable Cox regression formed the basis of the final prognostic model. With 53 disease-free survival events and nine regression parameters estimated in the multivariable Cox model, the resulting events-per-parameter ratio was approximately 5.9. Multicollinearity among variables included in the multivariable Cox model was formally assessed using variance inflation factors (VIFs) and tolerance values. No evidence of problematic multicollinearity was observed, with all VIF values below 3.0 (maximum VIF, 2.80) and all tolerance values above 0.35 (minimum tolerance, 0.357).

The discriminative performance of the prognostic model was quantified using Harrell’s C-index, and improvement across models was assessed using likelihood-ratio chi-square statistics. Internal validation was carried out using 1000 bootstrap resamples, allowing estimation of optimism-corrected model performance. Agreement between predicted and observed outcomes for the final model was examined using bootstrap-corrected calibration plots. All hypothesis tests were two-sided, with *p* < 0.05 defining statistical significance.

### 2.8. Ethics Statement

The study was approved by the Non-Interventional Clinical Research Ethics Committee of Van Training and Research Hospital, Van, Türkiye (Approval No: GOKAEK/2026-07-16; Approval Date: 17 July 2026). The requirement for informed consent was waived by the Ethics Committee due to the retrospective and non-interventional nature of the study, which involved review of existing medical records without direct patient contact or intervention. All patient data were anonymized prior to analysis, and confidentiality was strictly maintained throughout the study. The study was conducted in accordance with the ethical principles of the Declaration of Helsinki.

## 3. Results

### 3.1. Patient Characteristics

A total of 124 consecutive patients with locally advanced gastric adenocarcinoma who received neoadjuvant chemotherapy followed by surgery were screened for eligibility. After application of the predefined exclusion criteria, 109 patients who underwent curative-intent gastrectomy and had complete data for the variables required for the present analyses, together with adequate follow-up information, were included in the final analysis (Figure 1). During a median follow-up of 30.0 months (95% CI, 20.7–41.0), estimated using the reverse Kaplan–Meier method, 53 DFS events (48.6%) and 42 deaths (38.5%) occurred. The median disease-free survival for the entire cohort was 22.9 months (95% CI, 16.3–57.0). The median age was 62.0 years (range, 33–87 years), and 72 patients (66.1%) were male. A total gastrectomy was performed in 86 patients (78.9%), whereas 23 patients (21.1%) underwent subtotal gastrectomy. Standard D2 lymphadenectomy was performed in 97 patients (89.0%). Regarding the operative approach, 85 patients (78.0%) underwent open gastrectomy, and 24 (22.0%) underwent laparoscopic gastrectomy; no robotic gastrectomies were performed. Favorable pathological response (Becker TRG1–2) was achieved in 68 patients (62.4%), whereas 41 patients (37.6%) demonstrated minimal or no pathological response (TRG3). According to the proposed postoperative clinicopathological risk classification, 51 patients (46.8%) were classified as low risk, 25 (22.9%) as intermediate risk, and 33 (30.3%) as high risk. Baseline demographic, clinicopathological, treatment, pathological response, and follow-up characteristics are summarized in Table 1.

### 3.2. Clinicopathological Characteristics According to Becker Tumor Regression Grade

Baseline clinicopathological characteristics according to Becker tumor regression grade are presented in Table 2. Patients with a favorable pathological response were significantly more likely to present with lower pretreatment clinical T stage and clinical N stage (both *p* < 0.001). Histological grade also differed significantly across the three TRG categories (*p* < 0.001), whereas the Ki-67 index, pretreatment serum CEA, CA19-9, albumin levels, and neoadjuvant chemotherapy regimen showed no significant differences. Disease recurrence increased progressively with worsening pathological response, occurring in 13.0% of patients with TRG1, 42.2% of those with TRG2, and 75.6% of those with TRG3 (*p* < 0.001).

### 3.3. Predictors of Favorable Pathological Response

Univariable and multivariable logistic regression analyses evaluating predictors of favorable pathological response are summarized in Table 3. In univariable analyses, histological grade, pretreatment clinical T stage, and pretreatment clinical N stage were significantly associated with pathological response. After multivariable adjustment, pretreatment clinical T stage (cT4 vs. cT1–3; OR 0.033, 95% CI 0.004–0.304; *p* = 0.003) and pretreatment clinical N stage (cN2–3 vs. cN0–1; OR 0.112, 95% CI 0.038–0.332; *p* < 0.001) remained independent predictors of a reduced likelihood of achieving a favorable pathological response following neoadjuvant chemotherapy.

### 3.4. Prognostic Factors for Disease-Free Survival

The results of the univariable and multivariable Cox proportional hazards regression analyses are presented in Table 4. In the multivariable model, pretreatment clinical N stage (cN2–3 vs. cN0–1; HR 2.55, 95% CI 1.28–5.05; *p* = 0.007), Becker TRG3 (vs. TRG1; HR 5.16, 95% CI 1.35–19.69; *p* = 0.016), and log10-transformed CA19-9 (HR 1.44, 95% CI 1.01–2.05; *p* = 0.043) remained independent predictors of disease-free survival. These variables formed the basis for the final prognostic model.

### 3.5. Kaplan–Meier Survival Analyses

**Figure 2:**
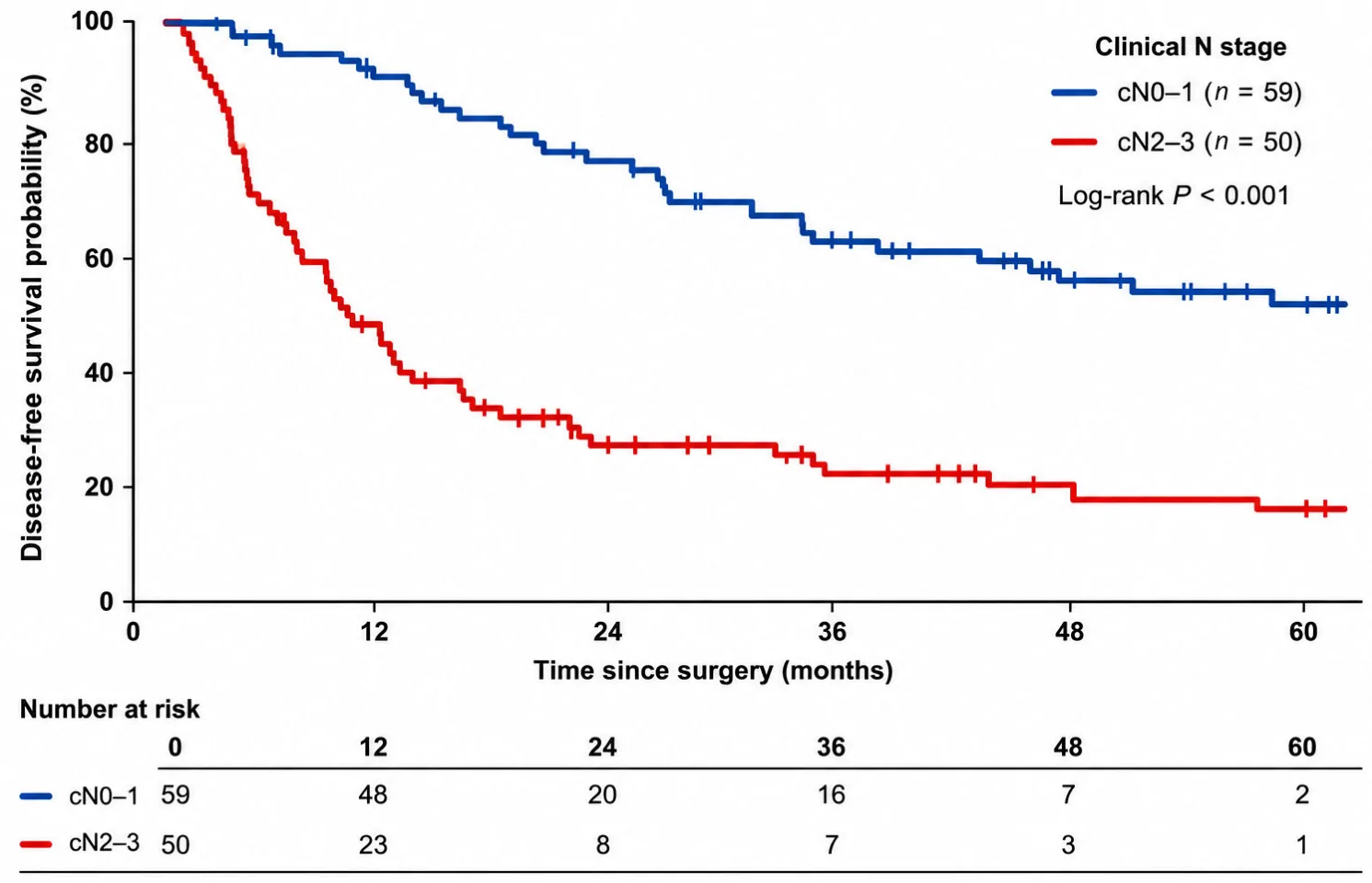
Kaplan–Meier curves for disease-free survival according to pretreatment clinical nodal stage.

**Figure 3:**
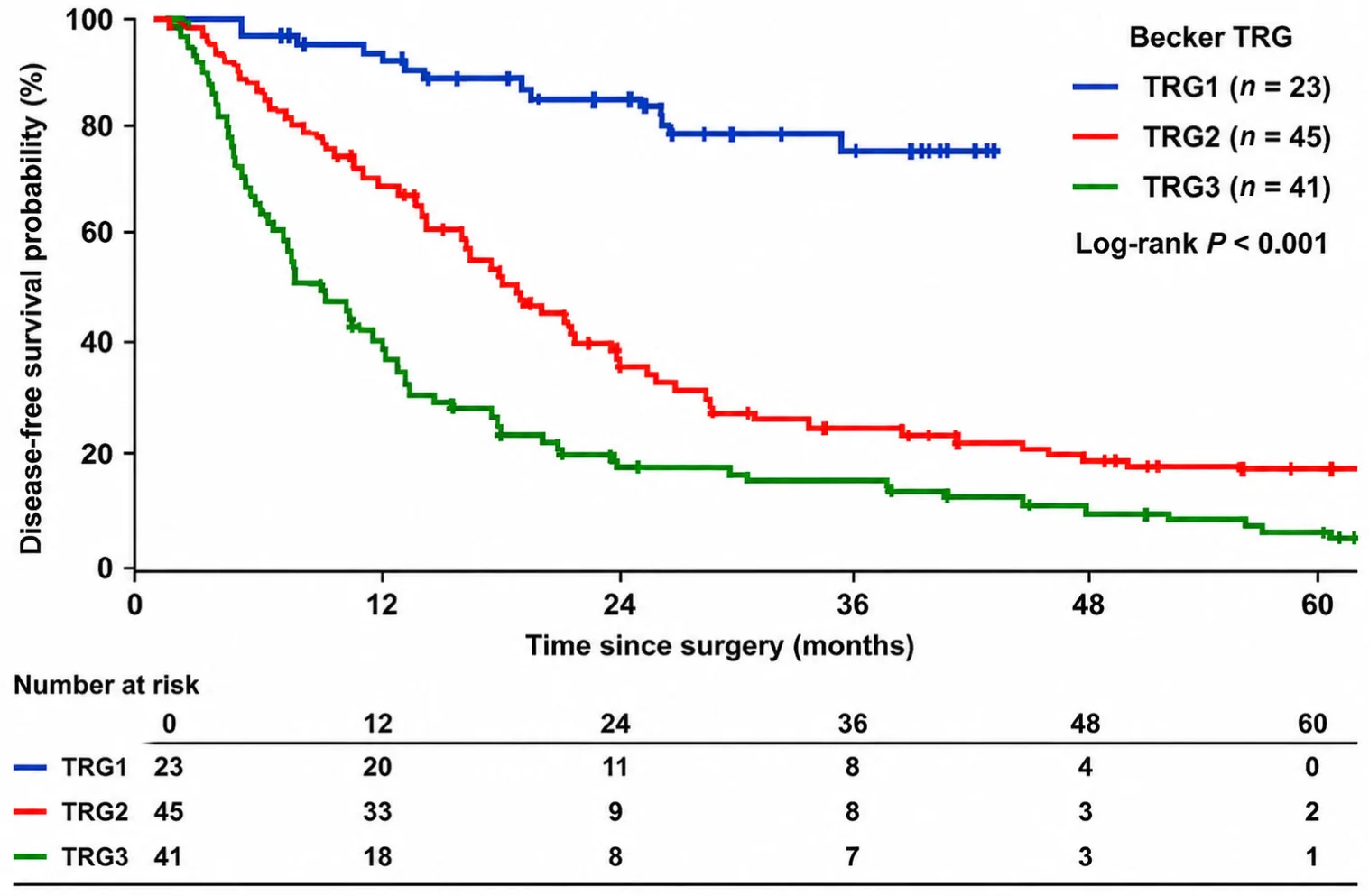
Kaplan–Meier curves for disease-free survival according to Becker tumor regression grade.

**Figure 4:**
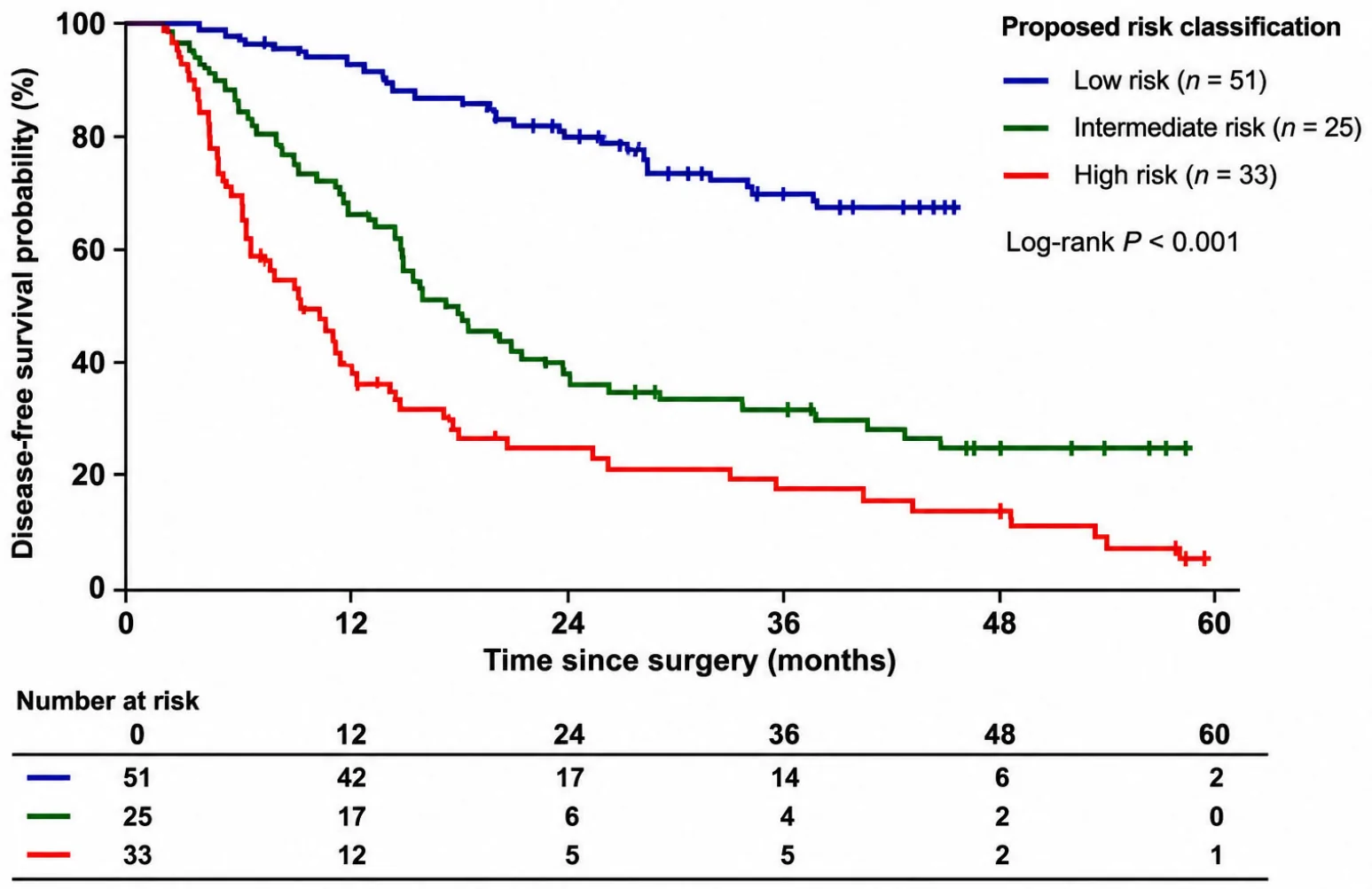
Kaplan–Meier curves for disease-free survival according to the proposed postoperative clinicopathological risk classification.

Kaplan–Meier survival analyses demonstrated significant differences in disease-free survival according to pretreatment clinical nodal stage (Figure 2), Becker tumor regression grade (Figure 3), and the proposed postoperative clinicopathological risk classification (Figure 4). The Kaplan–Meier curves showed progressively shorter disease-free survival with increasing pretreatment nodal burden, poorer pathological response, and higher postoperative clinicopathological risk.

### 3.6. Development of the Postoperative Clinicopathological Risk Classification

A practical postoperative clinicopathological risk classification based on pretreatment clinical nodal stage and Becker tumor regression grade was established to facilitate clinical risk stratification. The resulting classification identified 51 patients (46.8%) as low risk, 25 (22.9%) as intermediate risk, and 33 (30.3%) as high risk (Table 5). Disease recurrence occurred in 21.6%, 60.0%, and 81.8% of patients in the low-, intermediate-, and high-risk groups, respectively. Median DFS was not reached in the low-risk group, compared with 17.4 months in the intermediate-risk group and 9.3 months in the high-risk group, demonstrating progressively poorer outcomes across increasing risk categories.

### 3.7. Development and Internal Validation of the Prognostic Model

**Figure 5:**
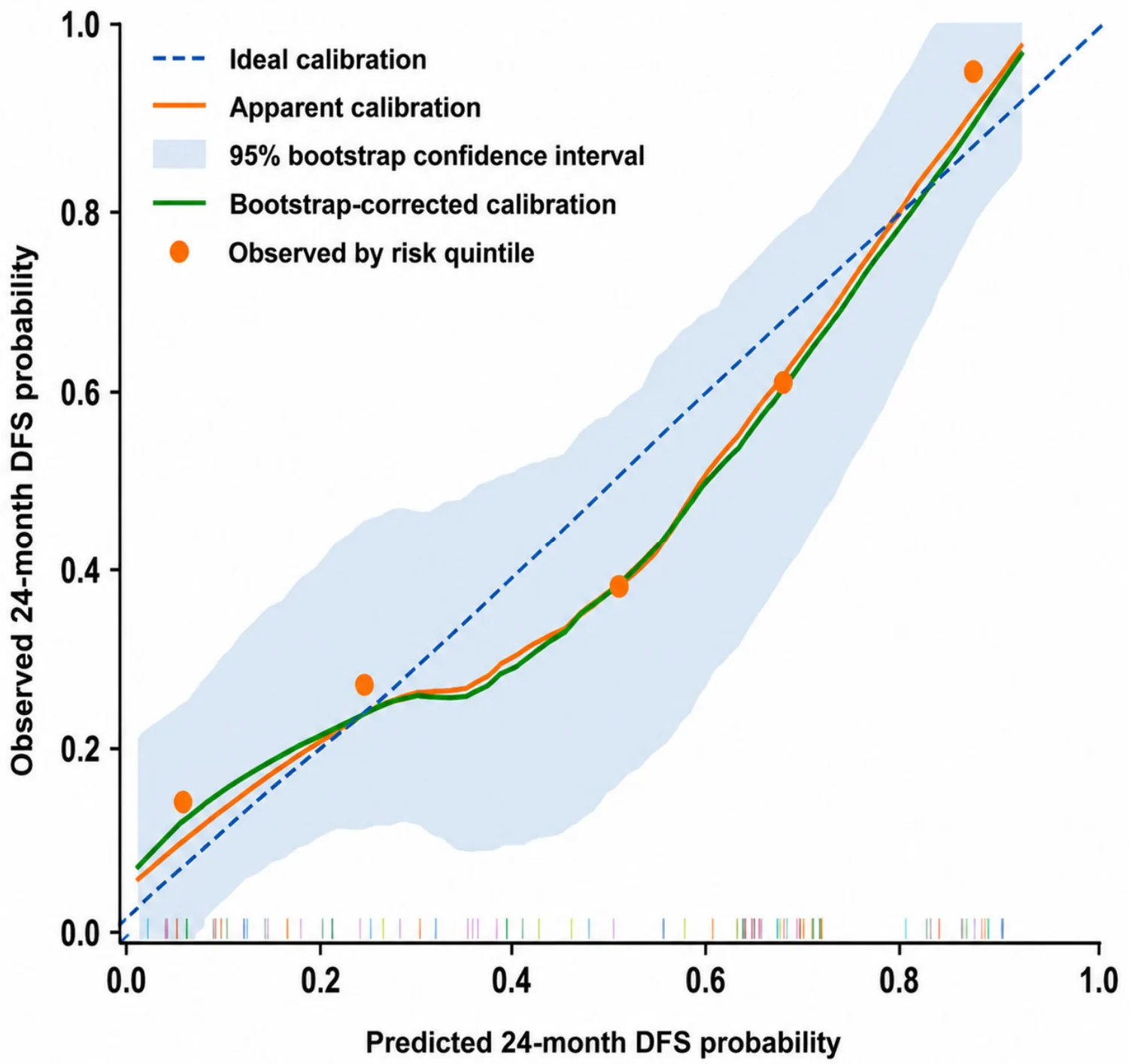
Bootstrap-corrected calibration plot for 24-month disease-free survival in the final prognostic model. The blue dashed line represents ideal calibration, the orange line represents apparent calibration, the green line represents bootstrap-corrected calibration, the light-blue shaded area represents the 95% bootstrap confidence in-terval, and the orange points represent observed probabilities by risk quintile.

Sequential model development demonstrated progressive improvement in predictive performance (Table 6). The optimism-corrected Harrell’s C-index increased from 0.697 for the model including pretreatment clinical N stage alone to 0.750 after incorporation of Becker tumor regression grade and further to 0.770 following the addition of log10-transformed CA19-9. Likelihood-ratio testing confirmed significant improvements in model performance. Bootstrap internal validation using 1000 resamples demonstrated minimal optimism. Furthermore, the bootstrap-corrected calibration plot showed good agreement between predicted and observed 24-month disease-free survival probabilities, indicating satisfactory calibration of the final prognostic model (Figure 5). Overall, these findings support the internal validity of the proposed prognostic model and provide a basis for future external validation.

## 4. Discussion

In this retrospective cohort study of patients with locally advanced gastric adenocarcinoma treated with neoadjuvant chemotherapy followed by curative gastrectomy, we developed and internally validated a clinicopathological prognostic model for disease-free survival integrating pretreatment clinical nodal stage, Becker tumor regression grade, and serum CA19-9. Compared with pretreatment clinical nodal stage alone, the final model achieved greater discriminatory performance, with the optimism-corrected Harrell’s C-index increasing from 0.697 to 0.770. We also established a simplified postoperative clinicopathological risk classification based on pretreatment clinical N stage and Becker tumor regression grade that effectively stratified patients into distinct recurrence-risk groups. Unlike previously published prognostic models, our approach combined sequential model development, bootstrap internal validation, and a simplified postoperative clinicopathological risk classification based exclusively on routinely available clinicopathological variables, thereby enhancing clinical interpretability while facilitating future external validation.

Pathological tumor regression following neoadjuvant chemotherapy is a well-established indicator of treatment response and long-term prognosis in gastric adenocarcinoma [6,7]. In our cohort, Becker tumor regression grade (TRG) remained an independent predictor of disease-free survival, and its incorporation into the prognostic model significantly improved discrimination beyond pretreatment clinical nodal stage alone. These findings reinforce the clinical value of pathological response assessment and support the integration of Becker TRG into postoperative prognostic evaluation after neoadjuvant therapy [7,8].

The prognostic significance of Becker TRG is biologically plausible because the extent of residual viable tumor reflects both intrinsic tumor biology and response to systemic therapy. Becker et al. first introduced this grading system and demonstrated its association with oncological outcomes after neoadjuvant chemotherapy [6]. Similarly, Urakawa et al. demonstrated in a Japanese cohort that histological response after neoadjuvant chemotherapy was associated with long-term prognosis in patients with gastric or gastroesophageal junction cancer [8]. The recent meta-analysis by Wu et al. showed that favorable tumor regression is consistently associated with improved survival and lower recurrence rates [7]. Our findings are consistent with these reports and further support that Becker TRG provides additional prognostic value when integrated with pretreatment clinical nodal stage and serum CA19-9 within an internally validated prognostic model.

Our findings further suggest that pathological response should not be interpreted in isolation. Although Becker TRG independently predicted disease-free survival, combining pathological response with pretreatment disease burden and serum biomarker status yielded greater prognostic discrimination. These findings are consistent with previous evidence [7,8] and further support the development of integrated clinicopathological prognostic models for individualized postoperative risk stratification.

Pretreatment clinical nodal stage was another key determinant of disease-free survival in our cohort. Patients with cN2–3 disease had a significantly higher risk of recurrence than those with cN0–1 disease, and pretreatment nodal stage remained independently associated with disease-free survival after adjustment for pathological response and other clinicopathological variables. Moreover, clinical nodal stage served as the foundation of the sequential prognostic models, with the addition of Becker TRG and serum CA19-9 providing incremental improvements in predictive performance. These findings emphasize the central role of pretreatment nodal burden in postoperative risk assessment.

The prognostic importance of pretreatment nodal status is well recognized in current gastric cancer guidelines and recent clinical studies. Both the Japanese Gastric Cancer Association and the ESMO Clinical Practice Guidelines identify clinical nodal involvement as a major determinant of treatment planning and long-term prognosis in patients receiving perioperative therapy [9,10]. More recently, Sun et al. demonstrated that the CT-based Node-RADS score improved prediction of survival outcomes compared with conventional clinical N staging, further highlighting the prognostic value of baseline nodal disease [13]. Together, these findings indicate that accurate pretreatment nodal assessment provides important prognostic information even after curative-intent surgery.

Our findings further suggest that pretreatment clinical nodal stage reflects not only anatomical disease extent but also baseline tumor burden and biological aggressiveness. Together with Becker tumor regression grade and serum CA19-9, pretreatment clinical nodal stage contributed to a more comprehensive postoperative prognostic assessment than that provided by any individual factor alone.

Serum CA19-9 was the third independent prognostic factor identified in our study and provided additional prognostic information beyond pretreatment clinical nodal stage and Becker tumor regression grade. Although the incremental contribution of CA19-9 was modest, its inclusion in the final multivariable model further improved predictive performance, increasing the optimism-corrected Harrell’s C-index from 0.750 to 0.770. These findings suggest that routinely available serum biomarkers can enhance postoperative prognostic assessment when integrated with clinicopathological variables.

The prognostic role of CA19-9 in gastric adenocarcinoma has been widely investigated. Elevated pretreatment CA19-9 levels have consistently been associated with advanced disease, greater tumor burden, and poorer long-term survival. Kambara et al. demonstrated in a Japanese cohort that elevated preoperative CA19-9 was independently associated with poorer relapse-free survival, supporting its prognostic relevance for postoperative recurrence-risk stratification in gastric cancer [11]. Importantly, the prognostic value of pretreatment tumor markers has also been demonstrated in a large European cohort from the CRITICS trial, in which elevated pretreatment CA19-9 was independently associated with worse survival in patients with resectable gastric cancer receiving perioperative treatment [15].

Our findings further support interpreting CA19-9 in combination with pretreatment clinical nodal stage and Becker tumor regression grade rather than as an isolated biomarker, reinforcing the value of integrated clinicopathological prognostic models for postoperative risk stratification.

A major strength of this study is the development and internal validation of a sequential clinicopathological prognostic model rather than the evaluation of isolated prognostic factors. Sequential incorporation of Becker tumor regression grade and serum CA19-9 into the baseline clinical nodal stage model yielded incremental improvements in predictive performance. Bootstrap internal validation demonstrated minimal optimism, while calibration analysis showed good agreement between predicted and observed disease-free survival probabilities, supporting the internal validity of the proposed model.

The growing emphasis on multivariable prognostic modeling reflects the recognition that postoperative outcomes cannot be fully explained by individual clinicopathological variables. Recent studies have similarly shown the value of integrating complementary predictors into prognostic models for postoperative risk stratification. Belia et al. externally validated the PERI-Gastric prediction models in an independent cohort of patients undergoing gastrectomy, supporting the importance of model validation when assessing postoperative recurrence risk [16]. Likewise, Aluariachy et al. developed a dynamic prognostic model for recurrence after curative surgery in patients with stage II–III gastric cancer and showed improved predictive performance by incorporating longitudinal biomarker information [17]. Reported discriminatory performance of prognostic models in gastric cancer varies considerably according to the study population, treatment setting, predicted endpoint, predictor selection, and validation strategy. In this context, the optimism-corrected C-index of 0.770 observed in the present study indicates useful discriminatory ability. Nevertheless, direct numerical comparison with previously published models should be interpreted cautiously, as existing models have been developed across heterogeneous clinical settings and frequently incorporate different postoperative, imaging-derived, or longitudinal predictors [15,16,17]. Accordingly, the principal value of the present model should not be interpreted as demonstrating superiority over existing prognostic tools, but rather as achieving clinically meaningful discrimination using a parsimonious combination of routinely available pretreatment, pathological-response, and serum biomarker variables. Compared with previously published models, our approach offers several practical advantages. It incorporates only routinely available pretreatment clinical nodal stage, Becker tumor regression grade, and serum CA19-9, making it potentially suitable for future clinical application without requiring advanced imaging, molecular biomarkers, or artificial intelligence-based algorithms. Moreover, the sequential modeling strategy clearly demonstrates the incremental prognostic contribution of each variable, facilitating clinical interpretation and future external validation.

Beyond its prognostic value, the proposed clinicopathological model may have important implications for postoperative clinical decision-making. Identifying patients at different risks of recurrence using routinely available clinicopathological variables may facilitate individualized postoperative surveillance and improve patient counseling following curative gastrectomy. Patients classified as high risk may benefit from closer radiological follow-up, more intensive surveillance, or enrollment in future clinical trials evaluating postoperative treatment intensification, whereas those in the low-risk group may be suitable for standard surveillance. In addition, communicating risk-group assignment may help clinicians discuss expected recurrence risk and the rationale for follow-up intensity with patients in a more structured manner. The identification of a high-risk subgroup may also help define populations for future studies evaluating intensified or novel postoperative therapeutic strategies. Although this study was not designed to guide treatment selection, the proposed risk classification provides a practical framework for postoperative risk stratification and may support personalized follow-up after external validation.

The present study has several methodological features that should be considered when interpreting the findings. The study population was restricted to patients who received neoadjuvant chemotherapy followed by curative-intent gastrectomy, providing a relatively uniform clinical setting for model development. The final model combines information obtained at different stages of the treatment pathway: pretreatment nodal status, pathological response to neoadjuvant therapy, and serum CA19-9. Importantly, all three variables are readily available in routine clinical practice. Model performance was examined through sequential comparisons, Harrell’s C-index, likelihood-ratio testing, calibration, and internal validation with 1000 bootstrap resamples. We deliberately favored a limited number of clinically accessible predictors rather than constructing a more complex model. This approach may make the model easier to interpret and provides a practical basis for subsequent validation in independent patient cohorts. Finally, the proposed risk classification is simple, clinically interpretable, and based entirely on routinely available variables, facilitating future external validation and potential clinical implementation. Following independent external validation, the prognostic model could potentially be translated into a nomogram or web-based calculator to facilitate individualized risk estimation in clinical practice. Although the optimism-corrected Harrell’s C-index of 0.770 indicates good discriminative performance, future studies incorporating molecular biomarkers and external validation in independent multicenter cohorts may further improve predictive accuracy and strengthen the generalizability of the proposed prognostic model.

This study has several limitations that should be acknowledged. First, the retrospective design may have introduced inherent selection bias despite the inclusion of consecutive eligible patients. Second, the absence of external validation represents an important limitation of the present study. In addition, the single-center design and relatively limited sample size may restrict the generalizability of the findings. The model was evaluated internally with 1000 bootstrap resamples and showed acceptable discrimination and calibration; however, its performance remains to be confirmed in independent populations. Validation in larger, preferably multicenter cohorts will therefore be necessary before the model can be considered for routine clinical use. The number of DFS events was also relatively small compared with the number of variables initially considered for modeling. Consequently, some degree of overfitting cannot be excluded despite the low optimism observed after bootstrap correction. Molecular information, including microsatellite instability (MSI), HER2 status, PD-L1 expression, and circulating tumor DNA (ctDNA), was not consistently available during the study period and therefore could not be incorporated into the present model. Recent evidence indicates that postoperative ctDNA positivity is associated with recurrence risk and may serve as a marker of molecular residual disease in patients with resectable gastric cancer [18]. These biomarkers may capture biological characteristics and residual disease risk that are not fully represented by conventional clinicopathological variables. Similarly, radiomics- and artificial intelligence-derived imaging features may provide complementary information on tumor heterogeneity, treatment response, and prognostic assessment [19]. Future studies should investigate whether integrating these molecular and imaging-derived biomarkers with the routinely available predictors used in our model provides incremental prognostic value and improves individualized recurrence-risk estimation. In addition, a direct comparison with postoperative pathological staging based on ypN or ypTNM was not performed because standardized data for these variables were unavailable for a proportion of the cohort. Future external validation studies should evaluate the incremental prognostic value of the proposed model beyond standard pathological staging. Finally, because of the retrospective nature of the study, postoperative treatment strategies were not completely standardized and may have evolved during the study period. Despite these limitations, the homogeneous treatment approach based on neoadjuvant chemotherapy followed by curative gastrectomy, together with comprehensive clinicopathological characterization and robust statistical validation, strengthens the reliability of our findings.

## 5. Conclusions

In conclusion, we developed and internally validated a practical clinicopathological prognostic model for predicting disease-free survival in patients with locally advanced gastric adenocarcinoma treated with neoadjuvant chemotherapy followed by curative gastrectomy. Sequential integration of pretreatment clinical nodal stage, Becker tumor regression grade, and serum CA19-9 provided incremental improvements in prognostic discrimination compared with pretreatment clinical nodal stage alone. In addition, the simplified postoperative clinicopathological risk classification effectively stratified patients according to recurrence risk using routinely available clinicopathological variables. Together, these findings provide a practical framework for individualized postoperative risk stratification and surveillance. External validation in independent multicenter cohorts is warranted before routine clinical implementation.

## Figures and Tables

**Table 1:** Baseline demographic, clinicopathological, treatment, pathological response, and follow-up characteristics of the study cohort (*n* = 109).

Variable	Overall Cohort (*n* = 109)
Demographic characteristics	
Age, years	62.0 (33–87)
Sex	
Male	72 (66.1)
Female	37 (33.9)
ECOG performance status	
0	52 (47.7)
1	53 (48.6)
2	4 (3.7)
Clinicopathological characteristics	
Histological grade	
Grade 1	8 (7.3)
Grade 2	63 (57.8)
Grade 3	38 (34.9)
Pretreatment clinical T stage	
cT1	1 (0.9)
cT2	40 (36.7)
cT3	50 (45.9)
cT4	18 (16.5)
Pretreatment clinical N stage	
cN0	11 (10.1)
cN1	48 (44.0)
cN2	31 (28.4)
cN3	19 (17.4)
Treatment characteristics	
Neoadjuvant chemotherapy regimen	
FLOT	88 (80.7)
Non-FLOT regimen	21 (19.3)
Pathological response	
TRG1	23 (21.1)
TRG2	45 (41.3)
TRG3	41 (37.6)
Proposed clinicopathological risk classification	
Low risk	51 (46.8)
Intermediate risk	25 (22.9)
High risk	33 (30.3)
Follow-up and outcome characteristics	
DFS events	53 (48.6)
Deaths	42 (38.5)
Median follow-up, months (reverse Kaplan–Meier)	30.0 (95% CI, 20.7–41.0)
Median disease-free survival, months (Kaplan–Meier)	22.9 (95% CI, 16.3–57.0)

Footnote: Data are presented as median (range) or number (%), unless otherwise specified. Median follow-up was estimated using the reverse Kaplan–Meier method from the date of curative-intent gastrectomy to death or the last available clinical follow-up. Disease-free survival was defined as the time from curative-intent gastrectomy to the first documented disease recurrence or death from any cause, whichever occurred first. Patients without an event were censored at last follow-up. The proposed clinicopathological risk classification combined pretreatment clinical nodal stage and Becker tumor regression grade. ECOG, Eastern Cooperative Oncology Group; FLOT, fluorouracil, leucovorin, oxaliplatin, and docetaxel; TRG, tumor regression grade; DFS, disease-free survival; cT, clinical tumor stage; cN, clinical nodal stage; CI, confidence interval.

**Table 2:** Baseline clinicopathological characteristics according to Becker tumor regression grade.

Variable	TRG1 (*n* = 23)	TRG2 (*n* = 45)	TRG3 (*n* = 41)	*p* Value
Pretreatment clinical stage				
Clinical T stage				<0.001
cT1	1 (4.3)	0	0	
cT2	17 (73.9)	21 (46.7)	2 (4.9)	
cT3	5 (21.7)	23 (51.1)	22 (53.7)	
cT4	0	1 (2.2)	17 (41.5)	
Clinical N stage				<0.001
cN0	5 (21.7)	5 (11.1)	1 (2.4)	
cN1	15 (65.2)	26 (57.8)	7 (17.1)	
cN2	2 (8.7)	13 (28.9)	16 (39.0)	
cN3	1 (4.3)	1 (2.2)	17 (41.5)	
Tumor biology				
Histological grade				<0.001
Grade 1	3 (13.0)	4 (8.9)	1 (2.4)	
Grade 2	19 (82.6)	28 (62.2)	16 (39.0)	
Grade 3	1 (4.3)	13 (28.9)	24 (58.5)	
Laboratory variables				
Ki-67, %	60 (10–90)	40 (10–90)	50 (10–90)	0.247
CEA, ng/mL	2.9 (0.65–11.4)	3.1 (0.98–134.0)	4.1 (0.92–158.0)	0.113
CA19-9, U/mL	13.1 (2.0–283.1)	11.1 (2.0–1610.0)	12.2 (2.0–1200.0)	0.925
Albumin, g/L	41.0 (21.7–46.0)	39.0 (19.5–48.0)	38.6 (23.4–50.0)	0.739
Treatment and outcome				
FLOT regimen	20 (87.0)	38 (84.4)	30 (73.2)	0.365
Disease recurrence	3 (13.0)	19 (42.2)	31 (75.6)	<0.001

Footnote: Data are presented as median (range) or number (%), as appropriate. Comparisons among the three Becker tumor regression grade groups were performed using the Kruskal–Wallis test for continuous variables. Because several categorical contingency tables contained small expected cell counts, categorical variables were compared using the Fisher–Freeman–Halton exact test with Monte Carlo estimation. Becker TRG1 indicates complete or subtotal tumor regression, TRG2 indicates partial tumor regression, and TRG3 indicates minimal or no tumor regression. CEA, carcinoembryonic antigen; CA19-9, carbohydrate antigen 19-9; FLOT, fluorouracil, leucovorin, oxaliplatin, and docetaxel; TRG, tumor regression grade.

**Table 3:** Univariable and multivariable logistic regression analyses for predictors of favorable pathological response (Becker TRG1–2).

Variable	Univariable OR	95% CI	*p* Value	Multivariable OR	95% CI	*p* Value
Histological grade, Grade 3 vs. Grade 1–2	0.184	0.078–0.432	<0.001	0.648	0.212–1.982	0.446
Clinical T stage, cT4 vs. cT1–3	0.021	0.003–0.167	<0.001	0.033	0.004–0.304	0.003
Clinical N stage, cN2–3 vs. cN0–1	0.081	0.031–0.208	<0.001	0.112	0.038–0.332	<0.001
FLOT vs. non-FLOT regimen	2.127	0.812–5.572	0.125	—	—	—
Log10-transformed CEA	0.473	0.204–1.100	0.082	—	—	—
Ki-67, %	0.986	0.969–1.003	0.116	—	—	—
Log10-transformed CA19-9	0.821	0.490–1.377	0.455	—	—	—

Footnote: Favorable pathological response was defined as Becker tumor regression grade (TRG) 1–2, whereas TRG3 was considered minimal or no pathological response. Univariable logistic regression analyses were performed for all candidate variables. Histological grade, clinical T stage, and clinical N stage were entered simultaneously into the multivariable model because of their strong univariable associations and clinical relevance as pretreatment predictors of pathological response. The dependent outcome was favorable pathological response, defined as Becker TRG1–2 and coded as 1; TRG3 was coded as 0. Clinical T stage was dichotomized as cT4 versus cT1–3, clinical N stage as cN2–3 versus cN0–1, and histological grade as Grade 3 versus Grade 1–2. Odds ratios below 1 indicate a lower likelihood of achieving a favorable pathological response following neoadjuvant chemotherapy. OR, odds ratio; CI, confidence interval; CEA, carcinoembryonic antigen; CA19-9, carbohydrate antigen 19-9; FLOT, fluorouracil, leucovorin, oxaliplatin, and docetaxel.

**Table 4:** Univariable and multivariable Cox proportional hazards regression analyses for disease-free survival.

Variable	Univariable HR	95% CI	*p* Value	Multivariable HR	95% CI	*p* Value
Histological grade 3 vs. grade 1–2	3.05	1.77–5.26	<0.001	1.19	0.62–2.30	0.598
Clinical T stage, cT4 vs. cT1–3	1.96	1.04–3.67	0.036	0.66	0.31–1.41	0.285
Clinical N stage, cN2–3 vs. cN0–1	4.64	2.55–8.46	<0.001	2.55	1.28–5.05	0.007
Non-FLOT vs. FLOT regimen	1.73	0.94–3.19	0.079	1.18	0.61–2.27	0.618
TRG2 vs. TRG1	4.11	1.21–13.91	0.023	2.61	0.74–9.18	0.135
TRG3 vs. TRG1	10.08	3.07–33.05	<0.001	5.16	1.35–19.69	0.016
Log10-transformed CEA	2.64	1.62–4.32	<0.001	1.69	0.96–2.97	0.071
Log10-transformed CA19-9	1.65	1.19–2.28	0.003	1.44	1.01–2.05	0.043
Albumin, g/L	0.98	0.94–1.03	0.422	0.97	0.93–1.02	0.262

Footnote: Disease-free survival (DFS) was defined as the time from curative-intent gastrectomy to the first documented disease recurrence or death from any cause, whichever occurred first. Patients without an event were censored at the last follow-up. Univariable Cox proportional hazards regression analyses were performed for all candidate variables. Variables demonstrating potential associations in univariable analyses, together with clinically relevant covariates, were included in an exploratory multivariable Cox model. Clinical T stage was dichotomized as cT4 versus cT1–3, and clinical N stage as cN2–3 versus cN0–1. Becker TRG was included as a categorical variable with TRG1 as the reference category. CEA and CA19-9 were log10-transformed before analysis; therefore, their hazard ratios represent the effect associated with a tenfold increase in marker level. Hazard ratios (HRs) > 1 indicate an increased risk of disease recurrence or death. HR, hazard ratio; CI, confidence interval; TRG, tumor regression grade; CEA, carcinoembryonic antigen; CA19-9, carbohydrate antigen 19-9; FLOT, fluorouracil, leucovorin, oxaliplatin, and docetaxel.

**Table 5:** Clinical outcomes according to the proposed postoperative clinicopathological risk classification.

Risk Group	Definition	Patients, *n* (%)	Disease Recurrence, *n* (%)	Median DFS (Months)
Low risk	cN0–1 + TRG1–2	51 (46.8)	11 (21.6)	Not reached
Intermediate risk	cN0–1 + TRG3 or cN2–3 + TRG1–2	25 (22.9)	15 (60.0)	17.4
High risk	cN2–3 + TRG3	33 (30.3)	27 (81.8)	9.3

Footnote: The proposed postoperative clinicopathological risk classification was defined by combining pretreatment clinical nodal stage and Becker tumor regression grade. The low-risk group comprised patients with cN0–1 disease and Becker TRG1–2. The intermediate-risk group included patients with discordant clinicopathological features, defined as cN0–1 with TRG3 or cN2–3 with TRG1–2. The high-risk group comprised patients with cN2–3 disease and TRG3. Disease-free survival was calculated from the date of curative-intent gastrectomy to the first documented disease recurrence or death from any cause, whichever occurred first. Patients without an event were censored at the last follow-up. Median DFS was estimated using the Kaplan–Meier method. “Not reached” indicates that the median DFS was not reached during follow-up. cN, clinical nodal stage; TRG, Becker tumor regression grade; DFS, disease-free survival.

**Table 6:** Incremental prognostic performance and internal validation of sequential Cox models for disease-free survival.

Model	Variables Included	Apparent Harrell’s C-Index	Bootstrap 95% CI	Mean Optimism	Optimism-Corrected C-Index	Likelihood-Ratio χ^2^	*p* Value
Model 1	Clinical N stage	0.697	0.635–0.757	0.000	0.697	Reference	—
Model 2	Clinical N stage + Becker TRG	0.755	0.690–0.817	0.006	0.750	10.67 (df = 2)	0.0048
Model 3	Clinical N stage + Becker TRG + log10-transformed CA19-9	0.777	0.712–0.838	0.008	0.770	5.22 (df = 1)	0.0224

Footnote: Internal validation was performed using 1000 successful bootstrap resamples. Disease-free survival was calculated from the date of curative-intent gastrectomy to the first documented disease recurrence or death from any cause, whichever occurred first. Clinical N stage was dichotomized as cN0–1 versus cN2–3. Becker tumor regression grade was entered as a categorical variable, with TRG1 as the reference category. CA19-9 was transformed using the base-10 logarithm. Apparent Harrell’s C-index represents discrimination in the original development cohort. Mean optimism was estimated as the average difference between model performance in each bootstrap sample and performance when the bootstrap-derived model was applied to the original cohort. The optimism-corrected C-index was obtained by subtracting mean optimism from the apparent C-index. Bootstrap 95% confidence intervals were derived from the percentile distribution of bootstrap C-index estimates. Likelihood-ratio tests compared Model 2 with Model 1 and Model 3 with Model 2. TRG, tumor regression grade; CA19-9, carbohydrate antigen 19-9; CI, confidence interval; df, degrees of freedom.

## Data Availability

The datasets generated and/or analyzed during the current study are not publicly available because they contain patient-level clinical data but are available from the corresponding author on reasonable request, subject to institutional and ethical regulations.
